# Normal range and predictors of serum erythroferrone in infants

**DOI:** 10.1038/s41390-023-02594-2

**Published:** 2023-04-17

**Authors:** Fredrik Bäckström, Anna Chmielewska, Magnus Domellöf, Staffan K. Berglund

**Affiliations:** 1 Department of Clinical Sciences, Pediatrics, Umeå University, Umeå, Sweden; 2 Wallenberg Centre for Molecular Medicine (WCMM), Umeå University, Umeå, Sweden

## Abstract

**Background:**

Erythroferrone (ERFE) has been identified as a hepcidin-regulating hormone synthetized by erythroblasts correlating to the erythropoietic activity and the needs for iron substrate in bone marrow of adults. The present study aimed to assess the ERFE serum concentrations and its predictors in infants.

**Methods:**

ERFE was explored at 4 time points during the first year of life in 45 healthy, breastfed, normal birth weight (NBW) infants, and 136 marginally low birth weight infants (LBW, 2000–2500 g) receiving iron (*N* = 58) or placebo (*N* = 78) between 6 weeks and 6 months of age.

**Results:**

ERFE concentrations were low at birth, increasing gradually during the first year of life. In NBW infants, reference ranges (5th to 95th percentile) were at 6 weeks <0.005–0.99 ng/mL and at 12 months <0.005–33.7 ng/mL. ERFE was higher in LBW infants at 6 weeks but lower at 12 months compared to NBW and minimally affected by iron supplementation among LBW infants. Correlations of ERFE with erythropoietic and iron status markers were weak and inconsistent.

**Conclusions:**

The role of ERFE in the crosstalk of erythropoiesis and iron homeostasis remains unclear in infants and further studies on ERFE in infants and older children are warranted within the framework of the erythropoietin–ERFE–hepcidin axis.

**Impact:**

- Normal range of erythroferrone in healthy infants is described for the first time.
- Erythroferrone in infants lacks correlation to iron status and markers of erythropoiesis.
- The findings indicate differences in infant regulation of iron homeostasis as compared to adults.
- The findings point to a need to study infant erythropoiesis separately from its adult counterpart.
- The findings may have clinical impact on management strategies of iron-loading anemia in infancy.

## Introduction

Iron deficiency (ID) is the most common micronutrient deficiency and iron deficiency anemia (IDA) is considered one of the most important burdens of global health. Due to the rapid growth in relation to the iron stores and intake, infants are at particular risk of developing ID or IDA and interventions to limit this on a global basis are warranted.^1^ Both deficiency and overload can be clinically relevant for the developing infant and understanding the regulation of iron metabolism is therefore essential when optimizing nutritional recommendations and interventions. The key regulator of iron uptake and cellular release, hepcidin, is upregulated by iron overload and downregulated by iron depletion.^2^ However, several other factors influence hepcidin to orchestrate iron homeostasis, foremost inflammation and erythropoiesis.^3^

The existence of a feed-back mechanism from erythropoiesis to hepcidin expression had already previously been hypothesized when erythroferrone (ERFE) was identified in 2014.^4^ ERFE is a protein secreted from erythroblasts. It has been shown to suppress hepcidin, thereby increasing iron uptake, in several mouse model experiments, and this function has been corroborated in human studies.^5^ In adults, erythroblast transcription of ERFE is upregulated by erythropoietin (EPO). ERFE subsequently downregulates hepcidin, thus establishing a regulatory loop from tissue hypoxia to expanded erythropoiesis and increased iron uptake mediated via an EPO–ERFE–hepcidin axis.^6,7^ The normal reference level (median (IQR)) of ERFE for healthy adults has been determined to be 8 (4–15) ng/mL.^8^

The mechanisms governing the developing erythropoiesis of infancy is less well characterized.^1^ Several circumstances differ from adults, the most striking of which may be the sheer expansion of the body mass roughly tripling from birth to 12 months, and consequently increasing demands on erythropoiesis and iron uptake. The transition from the low-oxygen intrauterine environment with primarily extramedullary erythropoiesis also creates the backdrop for an extremely dynamic process, where the activity of erythropoiesis may not always be mirrored by dietary needs of iron. While there may certainly be clinical applications of this knowledge, the challenge of understanding infant erythropoiesis is still one of basic science. In particular, very little is known about the role of ERFE in this interplay and only a few previous infant human studies have been published.^9–13^

The primary aim of this study was to define normal range of ERFE in a cohort of healthy normal birth weight (NBW) infants and explore its dynamic during the first year of life. Secondary aims were to compare ERFE concentrations in NBW to a cohort of infants with marginally low birth weight (LBW), a well-known risk group of iron deficiency, and to assess the correlation of ERFE during infancy to other known determinants of erythropoiesis and iron metabolism.

## Methods

This was an observational study assessing stored serum samples from infants participating in two previous study cohorts.

### The NBW, breastfed infants: LIME cohort

The first set of samples included sera from Swedish normal birth weight (NBW), breastfed infants, originally included as a reference population in the LIME study (Swedish acronym), a randomized controlled infant formula intervention trial.^14^ Briefly, the breastfed reference infants were not assigned to any intervention and the original inclusion criteria were birth weight 2500–4500 g, gestational age at birth ≥37 weeks, absence of chronic illness and neonatal diagnosis likely to affect any iron status outcome, no previous blood transfusion or iron supplementation, and exclusive breastfeeding at the inclusion with the intention to exclusively breastfeed until 6 months of age. In the present study, the first 45 breastfed infants included between 2014 and 2017 in the LIME study were analyzed for ERFE as a healthy NBW control group to the LBW at-risk group with regards to iron deficiency, and as a cohort on which to base reference values for ERFE serum concentrations. The LIME study was approved by the Regional Ethical Review Board in Umeå and registered at clinicaltrials.gov (NCT02103205).

### The LBW infants: JOHN cohort

The second set of samples included stored serum from Swedish marginally LBW infants (2000–2500 g), previously included in a randomized controlled trial of iron supplements, the JOHN study (Swedish acronym).^15^ Briefly, 285 LBW infants were included between 2004 and 2007 and randomized to receive placebo (*n* = 95), or iron supplementation at doses 1 mg (*n* = 95) or 2 mg/kg/day (*n* = 95) from 6 weeks to 6 months of age. Inclusion criteria were birth weight 2000–2500 g, no chronic diseases diagnosed at inclusion, and no previous blood transfusion or iron supplementation. Exclusion criteria in the present analyses were infants randomized to 1 mg/kg/day (*n* = 95), hematological disorder diagnosed during the study (*n* = 1), drop out from original study before 6 months of age (*n* = 17), poor compliance to the intervention (*n* = 21), infants unblinded from the randomized trial due to iron deficiency anemia (*n* = 15). Remaining LBW infants, randomized to placebo (LBW/no iron, *n* = 78) and 2 mg/kg/day (LBW/iron, *n* = 58), were analyzed for ERFE in the present secondary study. The JOHN study was approved by the Ethical Review Board in Umeå and registered at clinicaltrials.gov (NCT00558454). As previously published from the JOHN study, the placebo treated group had a high prevalence of ID at 6 months of age and thereby represents a high-risk population for iron depletion, while the iron supplemented LBW infants had an overall low ID prevalence.^15^

### Data collection and laboratory analyses

As a part of the original study design for the LIME study and the JOHN study, a large set of background and baseline characteristics were collected at inclusion, using delivery records and parental questionnaires. Data included sex, birth weight, birth length, and gestational age at birth. Furthermore, included infants were assessed at the study clinic according to their original study design. Blood samples were drawn from NBW infants (LIME study) at 6 weeks (inclusion), 4 months, 6 months and 12 months of age and blood samples from the two LBW groups (JOHN study) were drawn at 6 weeks (inclusion), 12 weeks, 6 months, and 12 months of age. At each visit, anthropometric measures were recorded, and blood samples were drawn for both immediate analyses of blood counts (including hemoglobin [Hb] and reticulocytes) and stored in multiple tubes as centrifuged serum at −80 degrees Celsius. As previously reported in both original studies, stored serum was later analyzed for iron status including ferritin, transferrin saturation (TS), transferrin receptor (TfR), and hepcidin.^14,15^

For this secondary project, stored serum was analyzed at the pediatric laboratory of Umeå University hospital for ERFE using enzyme-linked immunoassay (ELISA) (ERF-001, Intrinsic LifeSciences, La Jolla, CA). Before analysis, the serum samples were diluted 1:10 in treated human serum provided with the kit. All samples were analyzed in duplicates and samples with coefficient of variation above 15% or with levels falling outside the measuring range were re-assayed in dilution 1:5. This amendment to the test protocol was discussed and after data sharing confirmed as valid with the manufacturer. Samples with a level below the lowest standard measure (<0.005 ng/mL) were set to 0.005 ng/mL in statistical analyses.

### Statistical analyses

The original studies were powered based on primary outcomes and the present sample size was limited by the number of stored samples. All available samples from the three study groups were analyzed for ERFE and the pre-study assumption was that a clinically relevant effect size would be possible to detect. ERFE showed a skewed distribution, and all statistical analyses were performed using non-parametric tests. Descriptive data including median and percentiles 5, 25, 75, and 95 were used to assess the reference NBW population and normal range was defined as 5th to 95th percentile. Secondly, using Mann–Whitney *U*-test, ERFE was compared between NBW infants and the LBW infants (both subgroups combined) at 6 weeks, 6 months, and 12 months and between the two LBW groups at all four time points. Third, associations between ERFE and iron status and erythropoietic markers were assessed using Spearman rank correlation analyses. In addition, and due to a significant number of samples with non-detectable ERFE, the variables were also dichotomized according to detectable or non-detectable levels and further explored for predictors using logistic regression. In the regression models, lack of interaction was assumed, and calculations were performed for the pooled total study sample for samples collected at the same time point (6 weeks, 6 months, and 12 months).

## Results

**Fig. 1:**
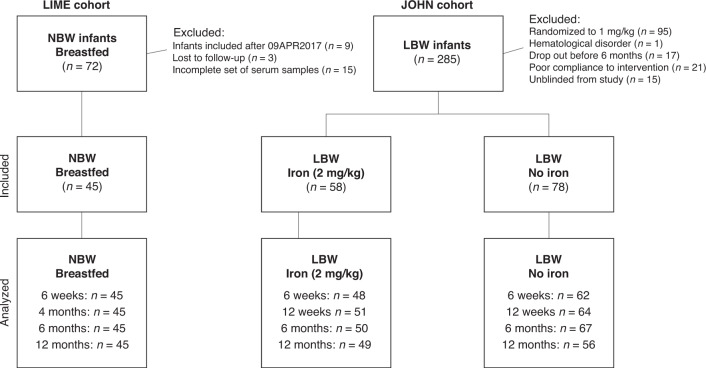
Trial profile. The study included 45 normal birth weight (NBW) breastfed infants originally participating as a reference group in the randomized trial LIME^14^ and 136 low birth weight (LBW) infants originally randomized to receive iron (2 mg/kg/day, *n* = 58) or placebo (*n* = 78) in the randomized controlled trial JOHN.^15^

**Fig. 2:**
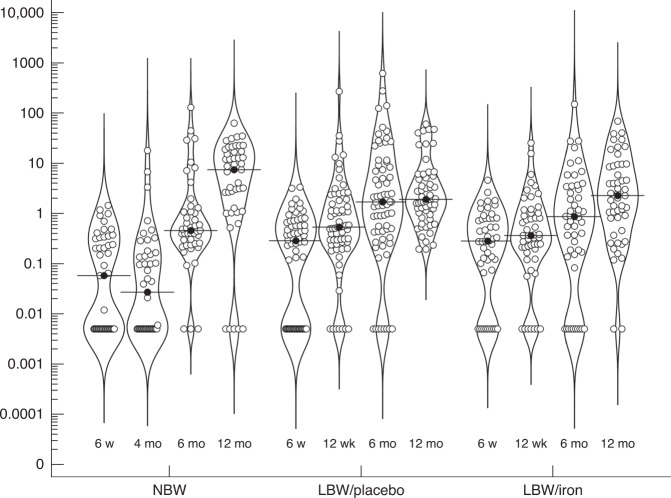
Violin plots for measures (ng/mL) of erythroferrone (ERFE) at four time points during the first year of life. Three groups are compared; breastfed normal birth weight controls (NBW) and low birth weight (LBW) infants supplemented with placebo or 2 mg/kg/day of iron between 6 weeks and 6 months of age. Horizontal line represents median.

ERFE was successfully measured in 151–163 infants at each of the 4 time points (Figs. 1 and 2). Background characteristics for included cases as well as results on iron results and hematological parameters previously analyzed are presented in Table 1. Of the analyzed samples, 54 (35%), 34 (21%), 21 (13%), and 7 (5%), respectively, at each time point were set to 0.005 ng/mL due to a result below detection limit. As illustrated in Fig. 2, the samples clustered into two groups, undetected samples (<0.005 ng/mL) and those with measurable values. There were no significant differences in any of the background variables between infants with detectable compared to undetectable ERFE at inclusion.

**Table 1:** Baseline characteristics and iron status in the included infants.

	NBW (*n* = 45)	LBW/placebo (*n* = 75)	LBW/iron (*n* = 58)
Girl, *n* (%)	25 (56%)	41 (55%)	25 (43%)
Preterm, *n* (%)	0 (0%)	38 (51%)	36 (62%)
Small for gestational age, *n* (%)	0 (0%)	32 (43%)	23 (40%)
Birth weight (kg)	3.57 (3.46–3.68)	2.29 (2.26–2.32)	2.30 (2.27–2.34)
Birth length (cm)	50.3 (49.8–50.9)	45.1 (44.8–45.5)	45.4 (45.0–45.7)
Gestational age (weeks)	40.0 (39.7–40.4)	36.6 (36.2–37.0)	36.2 (35.7–36.7)
Hemoglobin at 6 weeks (g/L)	117.9 (13.0)	108.2 (11.1)	107.1 (11.6)
Hemoglobin at 12 weeks/4 months^a^ (g/L)	113.4 (8.9)	107.2 (7.3)	107.8 (7.1)
Hemoglobin at 6 months (g/L)	114.7 (7.1)	114.0 (7.3)	122.3 (9.8)
Hemoglobin at 12 months (g/L)	114.9 (7.1)	117.6 (10.6)	117.6 (8.2)
Hepcidin at 6 weeks (ng/mL)	84.6 (31.9)	14.1 (5.1)	14.7 (20.9)
Hepcidin at 12 weeks/4 months^a^ (ng/mL)	42.7 (27.4)	22.1 (48.0)	23.7 (14.6)
Hepcidin at 6 months (ng/mL)	32.7 (27.1)	20.0 (19.6)	25.4 (17.8)
Hepcidin at 12 months (ng/mL)	N/A	16.5 (8.7)	13.2 (7.8)
Reticulocytes at 6 weeks (×10^9^/L)	47.6 (18.2)	62.4 (23.8)	64.4 (22.0)
Reticulocytes at 12 weeks/4 months^a^ (×10^9^/L)	46.4 (14.9)	68.8 (20.6)	77.0 (19.8)
Reticulocytes at 6 months (×10^9^/L)	42.8 (15.8)	57.2 (18.6)	56.1 (18.4)
Reticulocytes at 12 months (×10^9^/L)	44.4 (15.8)	57.0 (22.5)	41.3 (12.1)
Iron deficiency at 6 months, *n* (%)	5 (11.1%)	23 (31.5%)	1 (1.8%)

Values are *n* (%), mean (95% CI), or mean (SD).

*NBW* normal-birth-weight infants, *LBW/placebo* low-birth-weight infants (2000–2500 g) supplemented with placebo from 6 weeks to 6 months, *LBW/iron* low-birth-weight infants (2000–2500 g) supplemented with iron 2 mg/kg/day from 6 weeks to 6 months.

^a^NBW infants were assessed at 4 months of age and the LBW infants at 12 weeks of age.

Descriptive data of ERFE in NBW infants at 4 time points over the first year of life is presented in Table 2. Reference ranges (5th to 95th percentile) changed from <0.005–0.99 ng/mL at 6 weeks to <0.005–33.7 ng/mL at 12 months. In each group, respectively, ERFE showed an increasing trend during the first year of life (Fig. 2) and ERFE was significantly higher at 12 months compared to 6 weeks of age in all 3 groups (*p* < 0.001). Compared to NBW infants, LBW infants had significantly higher ERFE at 6 weeks (Median [5th; 95th percentile] was 0.28 [<0.005; 1.9] vs. 0.058 [<0.005; 1.12], *p* = 0.004) and significantly lower levels at 12 months of age (1.9 [0.17; 43] vs. 7.39 [<0.005; 34], *p* = 0.013). Between the two LBW groups, significantly higher ERFE was observed in the placebo group at 6 months of age (1.7 [<0.005; 133]) compared to iron supplemented infants (0.86 [<0.005; 27], *p* = 0.036), but no differences were observed at 6 weeks, 12 weeks, or 12 months.

**Table 2:** Descriptive data of ERFE (ng/mL) during infancy in 45 normal birth weight infants.

Age	5th Percentile	25th Percentile	Median	75th Percentile	95th Percentile
6 weeks	<0.005^a^	<0.005	0.06	0.33	0.99
4 months	<0.005	<0.005	0.03	0.15	3.33
6 months	<0.005	0.25	0.45	1.08	31.05
12 months	<0.005	1.27	7.39	17.22	33.67

^a^Of the analyzed samples, 54 (35%), 34 (21%), 21 (13%), and 7 (5%), respectively, at each time point were below detection limit (<0.005 ng/mL).

**Fig. 3:**
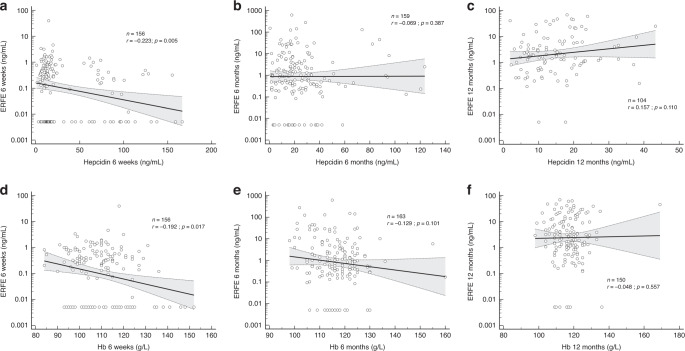
Scatter plots illustrating the correlation at 3 different time points during infancy of ERFE (ng/mL). **a**–**c** Hepcidin (ng/mL) and **d**–**f** Hb (g/L). Analyses included both NBW breastfed infants and LBW infants randomized to placebo or 2 mg/kg/day of iron, except for hepcidin at 12 months of age only available for LBW infants. Statistics presented from Spearman rank correlation analyses.

Univariate non-parametric linear regression analysis assessing the associations to markers of erythropoiesis and iron status is presented in Table 3. The results showed that some markers of erythropoiesis and iron metabolism correlated significantly to ERFE albeit in an inconsistent manner. However, correlations were weak as illustrated by correlation coefficients in Table 3 and graphically for Hb and hepcidin in Fig. 3. Corresponding logistic regression for univariate prediction of the dichotomized variable detectable/non-detectable ERFE is presented in Supplementary Table 1. Anthropometric data were analyzed similarly but stratified for NBW/LBW due to differing growth reference populations between the cohorts. No anthropometric predictors of ERFE were detected (data not shown).

**Table 3:** Univariate correlation between ERFE and indicators of erythropoiesis and iron status at three different time points using Spearman rank correlation coefficient.

	6 weeks			6 months			12 months		
	*R*	*p*	*N*	*R*	*p*	*N*	*R*	*p*	*N*
Hepcidin	**−0.223**^a^	**0.005**	**156**	−0.069	0.387	159	0.157	0.110	104
Hemoglobin	**−0.192**^b^	**0.017**	**156**	−0.129	0.101	163	−0.048	0.557	150
Ferritin	−0.135	0.098	152	−0.086	0.274	162	−0.019	0.818	151
MCV	0.049	0.541	156	−0.084	0.289	163	−0.105	0.202	150
Transferrin saturation	0.088	0.275	156	0.050	0.530	162	0.120	0.152	145
Transferrin receptor	0.137	0.090	155	**0.204**^a^	**0.009**	**163**	−0.036	0.716	104
Transferrin	−0.077	0.337	156	**0.160**^b^	**0.041**	**163**	0.001	0.988	145
Reticulocytes	−0.036	0.668	147	0.116	0.151	154	−0.181	0.156	62
EPO	0.031	0.746	111	0.069	0.460	118	N/A		

Bold values represents significant associations.

^a^Significant association (*p* < 0.01).

^b^Significant association (*p* < 0.05).

## Discussion

This is to our knowledge the first study exploring ERFE longitudinally throughout the whole of infancy. Despite high erythropoietic activity, ERFE concentrations were generally very low during early infancy, approaching adult levels only at 12 months of age. We observed a minimal difference between infants exposed to iron supplementation as compared to placebo at 6 months of age, but the effect size was small and the direction was unexpected (higher in un-supplemented infants). Furthermore, there was a lack of strong associations to traditional indicators of iron status and erythropoiesis, including EPO and hepcidin.

Little is known about the physiological mechanisms behind iron homeostasis in infants and the discovery of ERFE as an important player in the homeostasis of adult erythropoiesis, may contribute important keys for understanding the corresponding infant physiology.^16,17^ The present study suggested a normal range of less than 1 ng/mL in newborns, slowly increasing during the first year of life. This is in line with previous data on cord blood ERFE.^11^ We found no previous studies on healthy subjects in later infancy to compare these results with, and rather few studies in children and adults. In the original publication on the human ERFE assay by Ganz et al., the levels of 58 healthy adult blood donors had a median (IQR) of 8 ng/mL (4–15 ng/mL).^8^ This is clearly in the vicinity of our finding of median (IQR) 7.4 ng/mL (1.3–17.2 ng/mL) in infants aged 12 months. Whether this convergence points to a stabilization of levels beyond the first year of life is an interesting avenue for future studies. Reference levels are also likely to be specific to different kits as indicated by the concentrations presented by El Gendy et al., which were more than 100-fold lower in children 1.5–15 years old than those of 1 year olds in our study.^9^ This clearly complicates the pooling of data from different studies and underscores the need for an international standard.

While there is a clear time-dependent dynamic to ERFE levels in the present study, the correlation to erythropoietic or iron-homeostatic parameters was weak and inconsistent, both when analyzed as continuous variables and the dichotomized variable non-detectable vs. detectable. Specifically, no clear or consistent correlation was observed either to hepcidin, the target of ERFE in the iron-homeostatic context, or to reticulocyte levels, which may serve as a surrogate variable for erythropoietic activity, the source of ERFE. A lack of correlation between ERFE and hepcidin in severely ill preterm newborns has been previously shown,^10,12^ and our findings may indicate that this is not a pathological circumstance but rather a reflection of normal infant physiology. There are several possible reasons for this lack of correlation. ERFE may serve other physiological functions in infants, confounding correlations. For instance, an identical gene product circulating in the blood has been described in adult muscle physiology (the anabolic hormone myonectin)^18^ and the possibility of the ERFE dynamic shown in the present study being at least partially reflective of some other developmental process must be considered. It may also support the viewpoint suggested by some that infant hematopoiesis is not yet regulated in the same manner as in adults or older children.^17^ In contrast, it has been shown previously in unwell neonates that ERFE does respond to exogenous EPO analog administration.^13^ This would suggest there is some integrity of the EPO-ERFE part of the EPO-ERFE-hepcidin axis, although the effect size seems very modest compared to what has been shown in adults.^8^ Furthermore, the data presented here does not indicate any trend over time towards a closer correlation between ERFE and iron-regulation predictors in older infants. El Gendy et al. did show such correlations in 66 children, 36 IDA patients and 30 healthy controls, of mean age 8.59 and 9.13 years, respectively.^9^ Further studies delineating ERFE development beyond the first year of life are warranted to give more insight as to when and how this occurs.

An unexpected observation was the twin-peaked distribution of ERFE values observed at all time points. Although decreasing with advancing age, a significant number of samples turned out below the detection limit. As to reasons for this we can only speculate. Most likely this relates to technical uncertainty in the assay at very low levels, overstating the discreteness of the peaks. Alternatively, we cannot rule out a true biologically active process turning the ERFE transcription on and off; a “switch”, hypothetically by, e.g., epigenetic events, but we judge this less probable. As for ERFE in general, no clear predictors for or typical profile of these two clusters were seen in background characteristics or in laboratory data using logistic regressions. It would, however, stand to reason that undetectable samples were less frequent at 12 months as median ERFE levels in general were increasing at that time point.

Comparing our reference population of NBW infants and the LBW groups, ERFE levels were in a similar range and showed a similar pattern of development. Although not explained by any iron or erythropoiesis marker, there was a statistically significant difference in ERFE concentrations between the NBW group and the LBW groups at 6 weeks. This may suggest an earlier onset of increased transcription of ERFE in LBW babies. Bahr et al. showed a similar significant difference in cord blood ERFE between healthy NBW term and LBW preterm (28–32 weeks) newborns,^13^ and it is notable that both our LBW groups had higher rates of preterm babies (36–37 weeks) compared to the NBW group, suggesting that the predictor to some extent may be gestational age as well as birth weight. Finally, the ERFE concentrations did not seem to be influenced much by the access to supplemented iron in the LBW cohort even though we previously observed significant differences in several iron status and erythropoietic markers between the same two groups.^19^ With a high prevalence of ID at 6 months, the LBW/placebo group represents a suitable model of iron-deficient infants compared to the iron supplemented peers. Since ERFE was minimally higher in the placebo group at 6 months when erythropoietic activity was lower, it further supports our observation of a weak or absent association between ERFE and erythropoietic activity at this age. Indeed, since infants after the age of about 2 months of age have a very high erythropoietic activity, they might have been expected to have higher ERFE levels than healthy adults, but we found the opposite. The high number of undetectably low values, in our opinion, underscores the low expression of ERFE compared to adults.

From a clinical viewpoint, there is an interest in ERFE as a target for pharmaceutical manipulation to prevent organ damage from uptake of excess iron, e.g., in iron-loading anemias, and some progress has been made in this effort.^20^ The results of this study, however, demonstrate that before such a therapeutical approach is considered in early childhood, further elucidation of infant erythropoiesis in general, and particularly the role of ERFE, is necessary.

## Conclusion

Despite high erythropoietic activity, circulating ERFE is low in early infancy reaching levels similar to adult levels at 1 year of age. Throughout infancy, there is a lack of correlation between ERFE and markers of erythropoiesis and iron homeostasis, suggesting limitations in extrapolating the adult EPO–ERFE–hepcidin axis as a mechanistic model or theoretical framework. Other mechanisms governing the interplay between erythropoiesis and iron uptake are likely to be present in infants and further basic research to better understand these mechanisms is needed to inform therapeutic strategies in infant hematology.

## Supplementary information

Supplemental table 1

## Data Availability

The datasets generated and analyzed during the current study are available from the corresponding author on reasonable request.
