# Supplementary material for: Normal range and predictors of serum erythroferrone in infants
Source: Pediatr Res. 2023 Apr 17;94(3):965–70. doi: 10.1038/s41390-023-02594-2 (PMC10444606; doi:10.1038/s41390-023-02594-2)
Supplement: Supplementary file 1 — Supplemental table 1 [file 41390_2023_2594_MOESM1_ESM.docx]

Supplemental table 1. Univariate logistic regression for predictors of elevated (≥0.005ng/mL) as compared to undetectable ERFE (<0.005ng/mL)

|  | 6 weeks | | | 6 months | | | 12 months | | |
| --- | --- | --- | --- | --- | --- | --- | --- | --- | --- |
|  | OR | *95%CI* | N | OR | *95%CI* | N | OR | *95%CI* | N |
| **Hepcidin** | **0.99*** | **0.98-1.00** | **155** | 1.00 | 0.98-1.03 | 159 | 1.01 | 0.85-1.21 | 104 |
| **Hemoglobin** | **0.96*** | **0.93-0.99** | **155** | 1.00 | 0.95-1.06 | 163 | 1.00 | 0.91-1.10 | 150 |
| **Ferritin** | **0.99*** | **0.99-1.00** | **151** | 1.00 | 0.98-1.01 | 162 | 0.97 | 0.94-1.01 | 151 |
| **MCV** | 1.00 | 0.93-1.06 | 155 | 1.01 | 0.90-1.14 | 163 | 0.93 | 0.75-1.15 | 150 |
| **Transferrin saturation** | 1.01 | 0.98-1.04 | 155 | 1.01 | 0.97-1.06 | 162 | **0.93** | **0.86-1.00** | **145** |
| **Transferrin receptor** | 1.09 | 0.89-1.32 | 155 | 0.95 | 0.87-1.04 | 163 | **5.44** | **1.04-28.6** | **103** |
| **Transferrin** | 0.52 | 0.15-1,76 | 155 | 1.30 | 0.43-3.91 | 163 | 11.9 | 0.74-192 | 145 |
| **Reticulocytes** | 1.00 | 0.98-1.01 | 146 | 1.00 | 0.98-1.03 | 154 | **0.92** | **0.85-0.99** | **62** |
| **EPO (log-transformed)** | 5.45 | 0.54-55.2 | 110 | 0.51 | 0.04-7.04 | 118 | N/A |  |  |

Data are Odds Ratio (OR) with 95% CI for predictor’s association to detectable ERFE (set to 1) vs non-detectable (set to 0). * Significant association (p<0.05)
